# Inflammatory Cell Distribution in Primary Merkel Cell Carcinoma

**DOI:** 10.3390/cancers6021047

**Published:** 2014-05-06

**Authors:** Rachel Wheat, Claudia Roberts, Tim Waterboer, Jane Steele, Jerry Marsden, Neil M. Steven, David J. Blackbourn

**Affiliations:** 1 School of Cancer Sciences and CR UK Centre for Cancer Research, College of Medical and Dental Sciences, University of Birmingham, Birmingham, B15 2TT, UK; E-Mails: r.l.wheat@bham.ac.uk (R.W.); Claudia.roberts@uhb.nhs.uk (C.R.); 2 University Hospitals Birmingham NHS Foundation Trust, New Queen Elizabeth Hospital Birmingham, Mindelsohn Way, Edgbaston, Birmingham, B15 2WB, UK; E-Mail: jerry.marsden@btinternet.com; 3 Infection and Cancer Program, DKFZ (German Cancer Research Centre), 69120 Heidelberg, Germany; E-Mail: T.Waterboer@Dkfz-Heidelberg.de; 4 Human Biomaterials Resource Centre, College of Medical and Dental Sciences, University of Birmingham, Birmingham, B15 2TT, UK; E-Mail: j.c.steele@bham.ac.uk; 5 Department of Microbial and Cellular Sciences, Faculty of Health and Medical Sciences, University of Surrey, Guildford, Surrey, GU2 7XH, UK

**Keywords:** Merkel cell carcinoma, lymphocyte, polyomavirus, immunohistochemistry, confocal microscopy

## Abstract

Merkel cell carcinoma (MCC) is an aggressive poorly differentiated neuroendocrine cutaneous carcinoma associated with older age, immunodeficiency and Merkel cell polyomavirus (MCPyV) integrated within malignant cells. The presence of intra-tumoural CD8+ lymphocytes reportedly predicts better MCC-specific survival. In this study, the distribution of inflammatory cells and properties of CD8+ T lymphocytes within 20 primary MCC specimens were characterised using immunohistochemistry and multicolour immunofluorescent staining coupled to confocal microscopy. CD8+ cells and CD68+ macrophages were identified in 19/20 primary MCC. CD20+ B cells were present in 5/10, CD4+ cells in 10/10 and FoxP3+ cells in 7/10 specimens. Only two specimens had almost no inflammatory cells. Within specimens, inflammatory cells followed the same patchy distribution, focused at the edge of sheets and nodules and, in some cases, more intense in trabecular areas. CD8+ cells were outside vessels on the edge of tumour. Those few within malignant sheets typically lined up in fine septa not contacting MCC cells expressing MCPyV large T antigen. The homeostatic chemokine CXCL12 was expressed outside malignant nodules whereas its receptor CXCR4 was identified within tumour but not on CD8+ cells. CD8+ cells lacked CXCR3 and granzyme B expression irrespective of location within stroma *versus* malignant nodules or of the intensity of the intra-tumoural infiltrate. In summary, diverse inflammatory cells were organised around the margin of malignant deposits suggesting response to aberrant signaling, but were unable to penetrate the tumour microenvironment itself to enable an immune response against malignant cells or their polyomavirus.

## 1. Introduction

Merkel cell carcinoma (MCC) is a cutaneous high grade neuroendocrine carcinoma that is locally invasive with high metastatic potential [1]. Over a 20 year period, the annual incidence rate in the United States has increased threefold, from 0.2 patients per 100,000 person-years (PY) in 1986, to 0.6 per 100,000 PY in 2006 [2]. Similarly, the 10-year age standardised incidence rate of MCC in England has increased from 0.1 to 0.2 per 100,000 PY (UK National Cancer Intelligence Network Data Briefing November 2011). The mortality rate of MCC is more than twice that of melanoma, being 46% at 5 years [3]. The prognosis is related to the stage of the disease at presentation, with survival more than 90% for T1a or local disease. However, MCC is an aggressive tumour, and median survival for patients with distant metastases is only 9 months [4]. Palliation of loco-regional and distant disease is often difficult to achieve, and development of more effective treatments necessitates a better understanding of the underlying biology.

There is evidence for a relationship of MCC to immune function. First, MCC occasionally regresses without treatment and in such cases, infiltration by immune cells has been described [5]. Second, MCC commonly occurs in people with conditions associated with immune dysfunction or suppression [5,6]. Third, the presence of Merkel cell polyomavirus (MCPyV) within MCC malignant cells has been reported in ~80% of MCC but may be even higher [7]. Serological studies show 64%–88% exposure to MCPyV among adults [8,9] and the virus is present at low frequency and in low copy number in non-malignant skin, non-MCC cancers and in respiratory secretions [10,11,12,13]. However, in patients with MCC, circulating antibody titres are particularly high, and specifically in MCC cells, MCPyV is clonally integrated and replication incompetent. The expression of viral genes potentially contributes tumour-specific antigenic targets for T cell responses that are currently being mapped [14].

The purpose of the present study was to characterise the distribution and functional properties of CD8+ T lymphocytes within the inflammatory microenvironment in primary MCC tumours. Formalin-fixed, paraffin-embedded (FFPE) archived tumours were studied, focusing solely on the primary tumour to limit the heterogeneity that might arise from within-patient malignant evolution. Patients with MCC commonly present and undergo diagnostic biopsy in outlying centres: the use of FFPE allowed access to a larger and more representative sample of patients with this rare skin cancer. Furthermore, tissue architecture is better preserved than is the case in cryo-preservation. These were analysed by conventional immunohistochemistry and, in many cases, by multicolour immune fluorescence and confocal microscopy (CFM). The latter technique permitted a high definition for individual cells and simultaneous testing of multiple biological parameters. Previously, CFM has not been extensively applied to fixed tissue for reasons of autofluorescence and difficulty in antigen retrieval. In this study we adopted and then developed a recently described protocol coupling antigen retrieval, indirect immunofluoresence and CFM [15] in order to determine the phenotype and microenvironment of CD8+ cells in relation to malignant cells and vessels.

## 2. Results and Discussion

### 2.1. Patients

Archived fixed primary MCC tissue was obtained and analysed for 20 patients (Table 1). None was known to be HIV-infected. Female to male ratio was 11:9, median age 76 years, with presentation on head and neck, limbs and trunk (*n* = 5, 14, and 1, respectively), 18 presenting with a primary only and two with regional involvement at diagnosis. MCPyV Large T antigen (LTA) was detected by immunohistochemistry (IHC) for 9/20 primary tumour samples. High titre circulating IgG for either MCPyV Viral Protein (VP) 1 or LTA was detected for 7/9 patients: the two with negative titres also had tumours negative for LTA on IHC but one patient with an LTA-negative tumour was serologically positive.

**Table 1:** Patient characteristics.

ID	Sex	Age at diagnosis	Site of primary tumour	MCC stage	Tumour MCPyV LT status	Serum IgG VP1/LTA	Survival time (m)
P16	F	73	Upper limb	I	+	nd	101 ^a^
P17	F	79	Lower limb	I	−	−	78
P21	F	79	Lower limb	I	−	nd	11
P22	M	77	Lower limb	II	+	nd	99
P25	F	84	Head&neck	III	−	nd	22
P26	F	78	Head&neck	II	+	nd	136
P27	F	84	Lower limb	II	−	nd	41
P30	M	87	Head&neck	II	−	nd	11
P43	M	82	Lower limb	II	−	+	14
P45	M	63	Lower limb	III	−	nd	10
P46	M	56	Lower limb	II	+	+	12
P50	F	90	Head&neck	I	−	−	3
P53	F	70	Upper limb	II	+	+	46 ^a^
P58	M	83	Upper limb	I/II	−	nd	19
P60	F	75	Upper limb	I/II	−	nd	40
P72	M	59	Upper limb	II	+	+	30
P74	F	72	Head&neck	I	−	nd	40
P77	M	66	Trunk	II	+	+	36 ^a^
P79	F	68	Upper limb	II	+	+	30 ^a^
P82	M	61	Upper limb	I	+	+	14

m, months; ^a^ time to last known clinical review.

The 20 primary specimens showed the characteristic histological appearances of MCC comprising monomorphic small blue cells with a typical nuclear chromatin pattern, scant cytoplasm and high mitotic index. The pathological appearances were typical of those described previously and well recognised [1]. All but three specimens comprised monotypic cellular sheets or nodules interrupted by broad relatively hypocellular septa containing fibrous and vascular structures. In addition, almost all specimens (17/20) demonstrated areas in which the tumour was broken up into small aggregates and delicate cords a few cell widths across, the so-called trabecular pattern, and, more unusually, transition into single tumour cells. The vascularity and inflammatory infiltrate within the 20 specimens is summarised in Table 2.

### 2.2. CD8+ Cell Phenotype

The primary purpose of this study was to explore the functional properties of CD8+ cells within MCC, because intra-tumoural CD8+ lymphocyte infiltration is reported to be independently associated with improved MCC-specific survival [16]. Conventional IHC and, for 13 specimens yielding sufficient sections, multicolour immune fluorescent staining coupled to CFM, were applied to serial sections.

**Figure 1:**
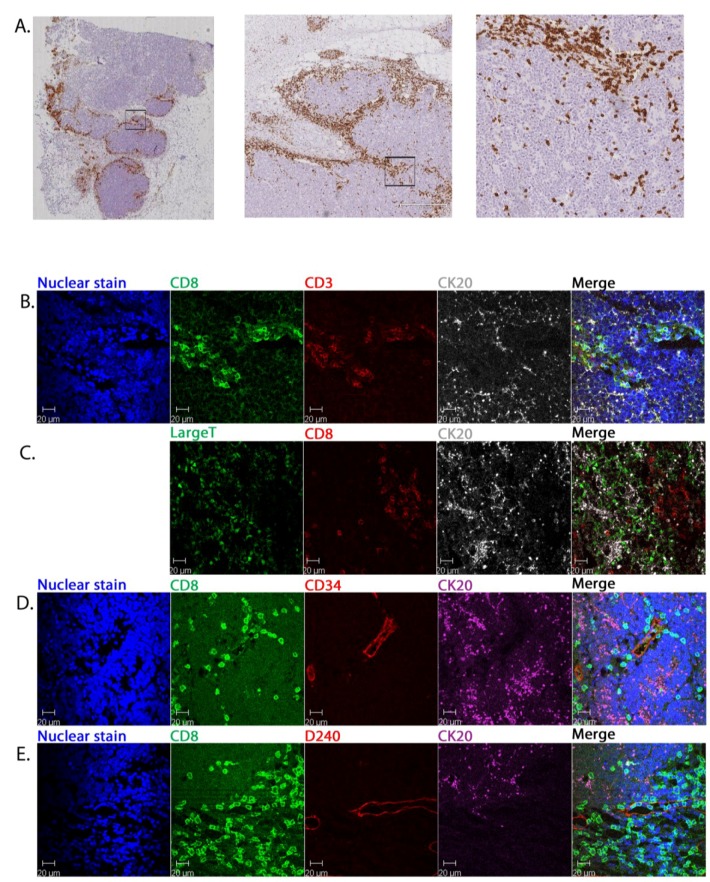
The distribution of CD8+ cells within primary MCC. IHC of primary MCC (patient P53) showing CD8+ cell distribution by conventional immunohistochemistry. The boxed regions show the area viewed at higher power in the adjacent panel to the right (**A**); Representative multicolour CFM images for the same patient indicating the relationship of CD8+ cells to other cells and structures: CD8+ CD3+ lymphocytes, CK20+ malignant cells (**B**); MCPyV LTA+ cells, CD8+ lymphocytes and CK20+ malignant cells (**C**); CD8+ lymphocytes, blood vessels lined by CD34+ endothelial cells, CK20+ malignant cells (**D**); CD8+ lymphocytes, lymphatic vessels lined by D240+ endothelial cells, CK20+ malignant cells (**E**). Nuclei are stained with bis-benzimide but on panel C this image is omitted for clarity. The merged image of all panels is shown at the far right.

Data from patient P53 (Figure 1) were representative of 11/13 primary MCC. On low power IHC of the whole specimen, CD8+ cells were seen to be distributed unevenly across the specimen and, where present, concentrated right on the margins of the tumour within the septa. CD8+ cells rarely appeared in contact with malignant cells (Figure 1A). High power view using multicolour CFM showed CD3+CD8+ cells clearly localised separately from the CK20+ MCC cells (Figure 1B). This tumour strongly expressed the potential viral immune target, MCPyV LTA. However, the CD8+ cells concentrated apart from the tumour cells expressing LTA, with only a limited number of CD8+ cells penetrating the tumour mass (Figure 1C). CD8+ cells had clearly extravasated; being identified within and around CD34+ blood (Figure 1D) and D240+ lymphatic (Figure 1E) vessels. The few CD8+ cells that had entered the tumour aggregates were typically arranged linearly (e.g., see Figure 1D, merged panel) suggesting migration along fine septa.

**Table 2:** Vascularity and immune cell abundance in primary MCC.

ID	Vessels within malignant aggregates		Inflammatory cell abundance and distribution throughout tumour specimen						Functional properties of inflammatory cells			CXCL12/CXCR4 expression	
	CD34 blood	D240 lymphatic	CD8	CD68	CD4	CD20	Fox P3	Dominant pattern of distribution	CXCR3	GrB	CD8+GrB+	CXCL12	CXCR4
P16	+	−	++	+	+	+	−/+	M/Tr					
P17	−/+	−	−/+	−/+	−	−	−	M/Tr	−/+	−/+	−		
P21	+	−	−	−	−/+	−	−/+	M/Tr					
P22	+	Focally +	+	+	+	+	−/+	M/Tr					
P25	+	−	+	+	+	−	−/+	M					
P26	+	−	+	+	+	+	−/+	M					
P27	+	−	−/+	+	−/+	−	−/+	M					
P30	+	−	+	+	+	+	−/+	M/Tr					
P43	+	−	−/+	−/+	+	−/+	−	M	−/+	−/+	−		
P45	−/+	−	+	+				M/Tr	−	+	−		
P46	+	−	−/+	+	−/+	−	−	M	+	+	−		
P50	Focally +	−	+	+				Tr	−/+	−/+	−		
P53	+	−	++	+				M	−/+	−/+	−	M	IT
P58	+	−	++	++				M/Tr/IT	−	−/+	−	M	IT
P60	Focally +	−	+	+				M/Tr	−/+	−/+	−		
P72	+	−/+	+	+				M	−/+	+	−	M	IT
P74	−/+	Focally +	+	+				M/Tr	−	−/+	−		
P77	+	−	+	+				M	−/+	−/+	−	M	IT
P79	−/+	−	+	+				M	−/+	−/+	−		
P82	+	−/+	++	+				M/Tr/IT	−/+	−/+	−		

Immune cell abundance is scored as abundant (++), present (+), rare (+/−) or absent (−). For vessels, focally positive means concentrated in broken trabecular areas of tumour. Dominant pattern of distribution: M, marginal: concentrated at edge of tumour in septa and penetrating tumour in smaller numbers within fine septa; Tr, trabecular: concentrated in areas of tumour break up into cords and small aggregates; IT, intra-tumoral: extensive infiltration within tumour mass away from margins. GrB, granzyme B. Empty cells mean not tested.

We next asked whether CD8+ cells were activated and responsive to inflammatory signaling by measuring expression of granzyme B and CXCR3. Granzyme B is a main component of cytotoxic granules that invokes target cell death [17]. CXCR3 is expressed on effector and memory T cells recruiting them to sites of inflammation in response to the IFN-γ inducible ligands CXCL9, CXCL10 and CXCL11 (see [18]). An accumulation of CXCR3+ T cells in tissue can serve as a marker for effectors responding to Th1 type inflammatory processes. Granzyme B+ cells were identified in 13/13 and CXCR3 in 10/13 specimens tested (Table 2). Granzyme B and CXCR3 expression largely coincided with each other: in 10/13 specimens many of the granzyme B+ cells were CXCR3+, whereas the remaining three specimens were CXCR3-negative (P45, P58, P74, Table 2). The frequency of these granzyme B+ cells was generally very low although three primary tumours contained areas with more intense infiltrates of these putatively cytotoxic cells (P45, P46 and P72, Table 2).

**Figure 2:**
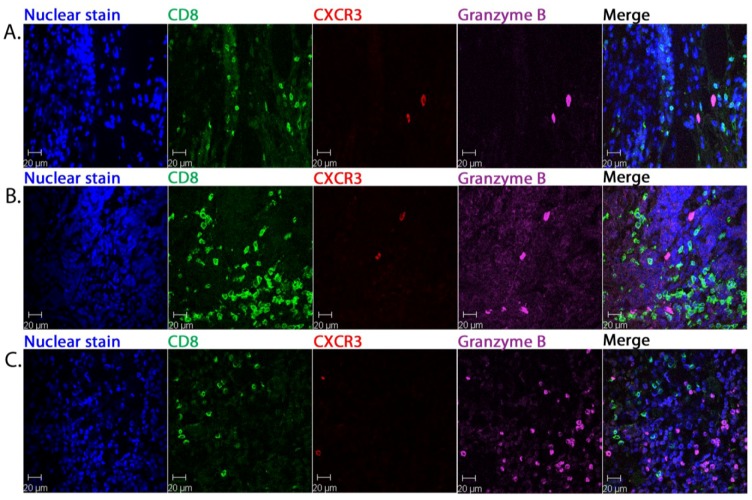
Detection of granzyme B and CXCR3 expression in relation to tumour associated CD8+ cells. Representative multicolour CFM images of primary MCC sections: CD8+ lymphocytes, CXCR3+ and granzyme B. Nuclei are stained with bis-benzimide. The merged image of all panels is shown at the far right. Images are from patient P53 (**A**), P82 (**B**) and P46 (**C**).

The granzyme B+ and CXCR3+ cells were consistently distinct from the CD8+ lymphocytes as illustrated for P53 (Figure 2A). Two specimens, P58 (data not shown) and P82 (Figure 2B) exhibited a variant pattern of CD8+ cell distribution with areas of intense CD8+ infiltration within tumour aggregates but these cells also were granzyme B and CXCR3-negative. This phenotype also applied to a further variant pattern with a very scanty CD8+ infiltrate throughout the tumour: even in patches within tumour where there was abundant granzyme B staining, the cytotoxic cells were CD8-negative (P46, Figure 2C).

These findings indicate that both intra-septal and tumour-infiltrating populations of CD8+ cells were functionally compromised and this held true irrespective of the intensity of the CD8+ infiltrate or the degree to which it penetrated into malignant aggregates.

**Figure 3:**
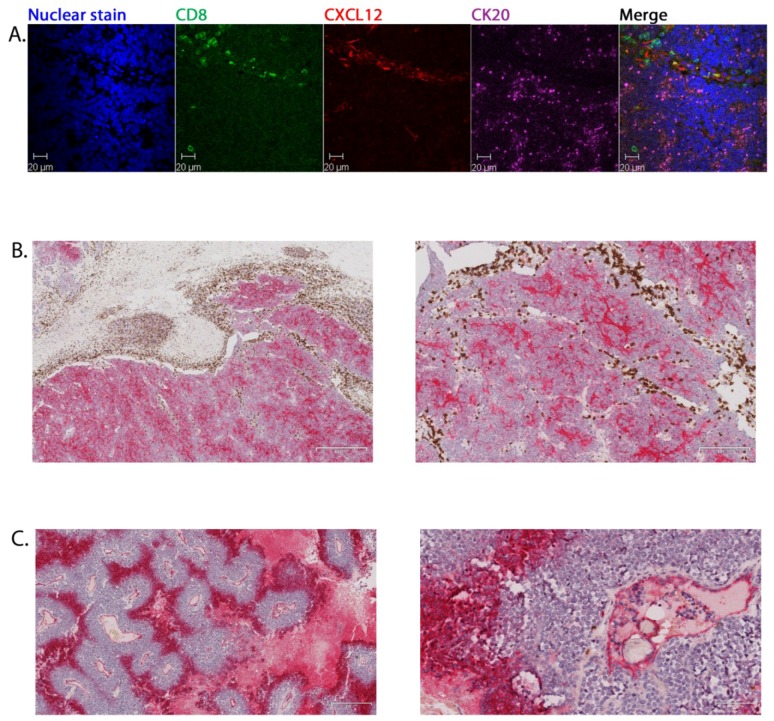
Expression of CXCL12 and CXCR4 within MCC. Representative multicolour CFM of primary MCC (patient P53): CD8+ lymphocytes and CXCL12, CK20+ malignant cells. Nuclei are stained with bis-benzimide. The merged image of all panels is shown at the far right (**A**); Dual colour immunohistochemistry of primary MCC sections: CXCR4 (red) and CD8+ (brown) cells, low (left) and medium magnification (right). Images from patient from patient P53 (**B**) and P77 (**C**).

CXCL12 is a homeostatic chemokine that coordinates cell trafficking and tissue organisation, including in the immune system. It is a potent chemoattractant for inflammatory cells and endothelial cells and is expressed in a wide range of cancers, predominantly by stromal cells. We therefore used multicolour CFM to ask whether CXCL12 might be expressed in MCC and determine its distribution. In samples from four patients, we demonstrated that CXCL12+ cells co-located as separate populations with CD8+ cells in the septal areas apart from the CK20+ tumour cells (Figure 3A).

CXCL12 acts by ligating its receptor, CXCR4. Therefore, we investigated the expression of CXCR4 in relation to malignant and CD8+ cells within primary MCC from four patients. Staining for one (P58) was inconclusive, but for the remaining three, dual-colour IHC showed clearly that it was malignant cells that were expressing CXCR4. The distribution was heterogeneous across the specimens, localised in areas separate to the septal and peripheral distribution of CD8+ cells (Figure 3B). Consequently, the stromal expression of the ligand, CXCL12, does not appear to explain retention of CD8+ cells. In some tumours, exemplified by P77 (Figure 3C), organised intense CXCR4 expression was evident, with MCC cell staining within cuffs radially distant from centrally located vasculature, possibly suggesting a relationship of CXCR4 expression to hypoxia. Endothelial cells were also CXCR4+, as demonstrated by staining of the cells lining the vascular structures (Figure 3C, right panel).

### 2.3. Tumour-Associated Inflammatory Cells

We next asked whether the presence and distribution of CD8+ cells was consistent with or discrepant from the pattern seen for a wider range of inflammatory cells, including CD68+ macrophages, CD4+ T cells, CD20+ B cells and FoxP3+ regulatory T cells, in relation to malignant and vascular structures within tumour (see Table 2).

**Figure 4:**
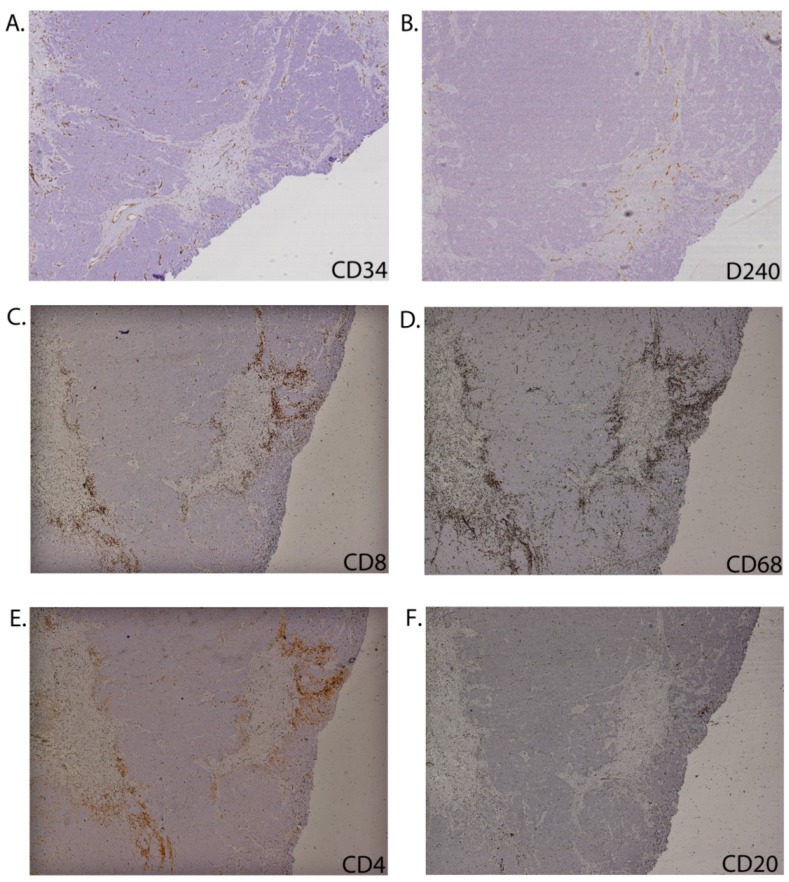
Distribution of vasculature and inflammatory cells in relation to the MCC primary tumour architecture. Immunohistochemistry of serial sections through primary MCC from patient 22 stained for CD34+ vascular endothelial cells (**A**); D240+ lymphovascular endothelial cells (**B**); CD8 (**C**); CD68 (**D**); CD4 (**E**); and CD20 (**F**).

Blood vessels lined by CD34+ endothelial cells were identifiable in all specimens. The most common pattern was an even distribution of vessels throughout the tumour structure, including both the tumour nodules and sheets, trabeculae and septa, sparing areas of necrosis. In six specimens, the vessels were concentrated in the septal areas and adjacent subcutaneous fat and either did not involve the tumour or were concentrated in trabecular regions of tumour broken into small aggregates and cords. The typical CD34+ vascular distribution is illustrated in Figure 4A. Lymphatic vessels were identified in 12 specimens, were sparse compared to CD34+ vessels and never identified within large tumour aggregates. Typical distribution is illustrated in Figure 4B. In two cases lymphatics appeared concentrated in the trabecular areas.

CD68+ macrophages and CD8+ cells were both identified in 19/20 primary MCC, albeit at low abundance in two of these. Four specimens contained abundant CD8+ cells. With few exceptions (P53 and P82), the number of CD68+ cells either paralleled or exceeded that for CD8+ cells. Representative staining is shown in Figure 4C,D, respectively.

Ten specimens were assessed for other inflammatory or immune cells: CD4+ cells were present in 9/10, CD20+ B cells in 5/10 (Figure 4E,F, respectively for representative staining), and FoxP3+ cells in 7/10 specimens (data not shown). Two tumours (P21, P43) were conspicuous in having rare inflammatory cells with CD4+ cells most prominent, whereas P27 contained CD68+ macrophages but almost no lymphocytes (Table 2).

The CD68+, CD4+, CD20+ and FoxP3+ cells broadly followed the same distribution as for CD8+ cells within each tumour (Table 2, see Dominant Pattern of Distribution column). These cells were unevenly distributed across the specimen, with some regions largely spared and others with a relatively intense presence around malignant aggregates. Where present, the CD68+ and CD4+ cells typically co-localised with CD8+ cells at the periphery and within septa directly at the interface with tumour. In much smaller numbers they were seen infiltrating into tumour sheets lined up in fine septa between malignant cells nests. B cells tended to form clusters peripheral to the tumour masses. CD68+ macrophages, but not CD8+ cells were found within areas of necrosis. In the two of our 20 primary cases (P58, P82) in which CD8+ cells observed infiltrating throughout tumour masses and potentially in contact with malignant cells, a similar pattern was also observed for CD68 and CD4+ cells.

Taken together, these IHC studies demonstrated that inflammatory cells were unevenly distributed across MCC specimens. Even in areas with a relatively high density of inflammatory cells, they were concentrated in septa along the margins of the tumour nodules and sheets. Penetration into tumour was most commonly limited to their being lined up within peri-marginal fine septa. This pattern of distribution suggested that the tumour-associated CD8+ cells shared common patterns of interactions with other inflammatory cell types, stromal components and the cancer itself.

## 3. Experimental

### 3.1. Patients and Samples

The National Research Ethics Committee East Midlands (08/H0405/59) approved the work. Anonymised clinical data and surplus pathological material were accessed for patients diagnosed with MCC and staging confirmed by the regional Skin Specialist Multidisciplinary Team in University Hospital Birmingham NHSF Trust. Median survival was estimated by the Kaplan-Meier method (Graphpad Prism 5.1). For a sub-group recruited prospectively, consent was obtained to acquire blood samples.

### 3.2. Haematoxylin and Eosin (H&E) Staining

FFPE sections from primary MCC tumours were cut at 4 µm, mounted, heated (60 °C, 10 min), deparaffinised, rehydrated in alcohol and rinsed in water before H&E staining.

### 3.3. Conventional Immunohistochemistry (IHC)

FFPE MCC tissue sections were prepared as described for H&E staining. They were then prepared and stained automatically (Dako Flex Ready to Use, Dako Denmark A/S, DK-2600 Glostrup, Denmark), counterstained using Mayers Haemalum (VWR 350604T, VWR International Ltd., Lutterworth, UK), dehydrated, cleared and mounted. Primary antibodies used for the conventional IHC were the same as for the multicolour immunohistochemistry (see below), but some were used at different dilutions in blocking buffer: CD8 Novocastra 1:50 (Leica Biosystems, Newcastle Upon Tyne, UK) and MCPyV LTA 1:20 (CM2B4; a kind gift of Yuan Chang and Patrick Moore, University of Pittsburgh Cancer Institute, PA, USA and available commercially as sc-136172 from Santa Cruz Biotechnology, Inc., Dallas, TX, USA; see below). Other antibodies included: CD4 (NCL-L-CD4-368 Novocastra, dilution 1:80); CD68 (M0876 Dako, dilution 1:200); CD20 (M0755 Dako, dilution 1:800); FOXP3 (Ab22510 Abcam, dilution 1:25, Abcam, Cambridge, UK).

### 3.4. Multicolour Immunohistochemistry

FFPE MCC tissue sections prepared as described for H&E staining were incubated at 60 °C for 30 min, deparaffinised in Histo-Clear (National Diagnostics HS-200, Geneflow Ltd., Lichfield, UK), cleared in absolute ethanol and washed briefly in tap water. Antigen retrieval treatment was by heating to 99 °C in Wax Capture (W-Cap) TEC Buffer pH8.0 (Bio-Optica 15-6315/F, Bio-Optica Milano SpA, Milan, Italy) for 40 min. Blocking was by incubating with 20% heat inactivated normal goat serum/0.1% BSA/PBS (20 min, room temperature) in a humidified chamber. Primary monoclonal antibodies, diluted in blocking buffer were: CD8 either from Novocastra (NCL-CD8-4B11 Novocastra, dilution 1:200) or Abcam (ab17147 Abcam, dilution 1:100); MCPyV LTA CM2B4 (sc-136172 Santa Cruz Biotechnology, dilution 1:125); CK20 Clone Ks20.8 (M7019 Dako, dilution 1:50); D2-40 (ab52092 Abcam, used at 1×); CD34 Class II (M 7165 Dako, dilution 1:100); CXCR3 (R&D MAB160, dilution 1:4000, R&D Systems Europe Ltd., Abingdon, UK); CXCL12 (R&D MAB350, dilution 1:20). The granzyme B antibody was rabbit polyclonal (ab4059 Abcam; dilution 1:50).

Antibodies were incubated with tissue overnight in a humidified chamber at 4 °C. An isotype-matched irrelevant antibody served as a negative control. Isotype control antibodies were: Sigma M5284 (for CD8 (Abcam), D2-40, and CD34); R&D Systems MAB004 for CD8 (Novocastra) and MCPyV LTA; Dako X0943 for CK20; R&D Systems MAB002 for CXCR3. Rabbit polyclonal isotype antibody (Abcam ab37415) matched the granzyme B primary antibody.

After primary antibody incubation, sections were washed and incubated with appropriate secondary antibodies, raised in goat. Each was labelled with a different fluorochrome, depending on the specificity of the antibody (Alexa488 IgG_2B_, Alexa633 IgG_2A_, Alexa633 H+L, all from Invitrogen (Molecular Probes, Inc., Eugene, OR, USA); TRITC IgG_1_, Southern Biotech, Cambridge Bioscience Ltd., Cambridge, UK). Nuclear counterstaining was with bis-benzimide (Invitrogen H21491). All slides were mounted with Immu-Mount (Thermo Scientific Shandon 9990402, Fisher Scientific UK Ltd., Loughborough, UK) and images captured with a Zeiss LSM 510 confocal laser scanning microscope (Carl Zeiss Ltd., Cambridge, UK).

### 3.5. Dual-Colour Immunohistochemistry

Automated one-step dual staining was performed with the Chromoplex 1 Dual Detection for BOND system (Leica Biosystems) for CD8 and CXCR4 at high pH antigen retrieval (pH 9). The CD8 antibody was that used for conventional IHC (Novocastra NCL-CD8-4B11) at 1:50. The CXCR4 antibody was from Epitomics (3108-1) at 1:100 (Epitomics, Burlingame, CA, USA).

### 3.6. MCPyV Serology

Serum samples were tested for seroreactivity to MCPyV VP1 and LTA as described [19,20].

## 4. Conclusions

In many cases MCC is loco-regionally aggressive and disseminated disease carries a poor outlook with no treatment established as inducing durable responses. There is a need to develop and test new treatment modalities that can target locally invading and disseminating disease. An important therapeutic goal is to induce inflammation within MCC nodules to attract CD8+ cells capable of responding to viral and cellular antigens on malignant cells. Indeed, the current literature demonstrates strong signals for an interaction between immunity and MCC that might serve as a source of treatment targets. Thus, MCC frequently associates with immunosuppressive conditions [5,6] and spontaneous regression is widely reported [21,22,23,24]. Furthermore, within MCC, measurable immune parameters in tumour are of clinical significance. In 35 MCC lesions, immune-related transcriptome profiles correlated with better outcome. Validating these findings by IHC on 129 MCC patients revealed that intra-tumoural as opposed to peri-tumoural CD8+ cell infiltration predicted MCC survival [16]. Moreover, the presence of CD8+, CD3+ and even FoxP3+ lymphocytes in MCC also associated positively with survival [25]. The majority of cases of MCC harbours integrated genomes of the human polyomavirus, MCPyV [10,26] and both viral and oncogenic cellular antigens expressed in MCC contain sequences potentially targeted by CD4+ helper and CD8+ cytotoxic T lymphocytes [14,27].

The purpose of this project was to investigate the detailed distribution and functional properties of CD8+ cells *in situ* in primary MCC and to describe the broader inflammatory infiltrate. Our data identified CD8+ cells as exiting vessels and localising on the margins of tumour deposits with limited penetration amongst malignant cells (see below). A similar distribution of inflammatory cells has been reported in other types of human tumour, including pancreatic ductal adenocarcinoma [28], epithelial ovarian cancer [29] and colorectal cancer [30], in which additional patterns of tumour inflammatory cell infiltration correlate with improved patient survival.

Since granzyme B serves as a marker for T cells exhibiting cytolytic capability [17,31] and CXCR3 on effector and memory T lymphocytes can identify T_H_1-driven inflammation (see [18]), we sought the presence of these markers within primary MCC. In metastatic melanoma, CXCR3 expression on tumour-reactive CD8^+^ cells was positively correlated with survival [32]. Likewise, in a murine cutaneous tumour model, the recruitment of CXCR3-bearing effector cells by CXCL9 correlated positively with tumour rejection [33]. In renal cell carcinoma, CXCR3 may selectively recruit T cells to the tumour [34]. Therefore, we predicted co-expression of these markers on CD8+ cells if accumulation of activated cytotoxic cells was in response to T_H_1-driven inflammation. Indeed, granzyme B+ cells were identified in all MCC specimens and in most these same cells were CXCR3+ (granzyme B-negative CXCR3+ cells were not observed). Nevertheless, in the majority of cases these were sparse. In three cases, areas containing more abundant granzyme B cells were identified, typically but not exclusively overlapping with areas of CD8+ cells. Importantly, CD8^+^ cells were never identified as CXCR3 positive.

Taken together, these data indicate that the CD8+ cells within primary MCC are positionally compromised and functionally impaired, having dormant cytolytic capability. The lineage(s) of the granzyme B+ and CXCR3+ cells within MCC remains to be determined. However, the scarcity or absence of CXCR3+ cells in most cases means that T_H_1-mediated inflammation did not explain the accumulation of inflammatory cells in the stroma and at the tumour margin. Nor did it explain the variant pattern of extensive intra-tumoural CD8+ cell infiltration in two primary MCC in our cohort.

CXCL12 is a chemokine with pleiotropic functions, expressed in diverse organs and by a range of cells including stromal fibroblasts and endothelial cells. Its expression is strongly associated with progression in several cancers, e.g., ovarian [35], including through promoting the proliferation and migration of malignant cells expressing its receptor CXCR4. Indeed, circulating antigen-specific CD8+ lymphocytes constitutively express CXCR4 [36] and the chemokine ligand can recruit cytotoxic T cells to melanoma cells in organotypic culture [37]. Localised CXCL12-CXCR4 interactions can retain CD8+ T cells in certain settings of chronic inflammation [38]. However, CXCL12 invokes concentration-dependent bidirectional movement that includes repulsion of effector cells at high concentrations [39]. Beyond the tumour microenvironment, CXCL12 may play an important role in the pathogenesis of rheumatoid arthritis. In this setting, the loss of a gradient of CXCL12 is thought to retain T cells in the synovium, causing or exacerbating chronic inflammation [40].

In four MCC specimens analysed, multicolour CFM demonstrated CXCL12 expression in stromal and endothelial cells that co-located with CD8+ cells separate to tumour cells. However, CD8+ cells anywhere in the tumour did not express CXCR4, demonstrating that their distribution was not mediated by this interaction. Interestingly, MCC cells expressed this receptor, suggesting a mechanism of vascular invasion and metastasis. Expression was most prominent in a lobular pattern radially distributed away from vessels suggesting a role for hypoxia mediating up-regulation of CXCR4, as noted previously [41].

Looking more broadly at the inflammatory infiltrate within primary MCC, we identified not only CD8+ lymphocytes, but also CD68+ macrophages, CD4+ and scanty FoxP3+ putative regulatory T cells in the majority of primary MCC. Although FoxP3+ cells may play a role in suppressing anti-tumour responses, Sihto *et al.* correlated FoxP3+ tumour-associated lymphocytes with better prognosis [25]. Thus, their functional properties in this setting remain to be elucidated. Strikingly, many MCC contained a pronounced CD68+ cell presence, probably marking mature macrophages, which in some cases exceeded lymphocyte numbers and extended from the septa into the tumour. Macrophages are highly plastic, broadly extending from pro-inflammatory to immune suppressor phenotypes. Tumour associated macrophages in breast cancer, for example, can promote tumor growth, angiogenesis, metastasis, remodelling of the extra-cellular matrix and immune evasion. Increased CD68+ infiltration in breast cancer is associated with poorer outcome [42]. Therefore there is a strong case for investigating the functional properties of this population and considering these as a therapeutic target.

Within each specimen, the tumour-associated inflammatory cells tended to co-locate densely in some regions, sparing other architecturally similar areas. Where present, the inflammatory cells typically were right at the margin of malignant nodules and in connective tissue separating tumour trabeculae. Smaller numbers of inflammatory cells were commonly observed, by both conventional microscopy and multicolour CFM (CD8+ cells only), extending from the margin into the nodules arranged linearly within fine septa and not among or in contact with malignant cells. CD34+ blood vessels were typically distributed throughout tumours, in malignant nodules, sheets and septa, or in a minority of cases, concentrated in septa. Lymphatics were sparse. The dominant distribution of inflammatory cells along the tumour margins was different to that of vessels and detailed examination by multicolour CFM showed CD8+ cells within septa, but outside both blood and lymphatic vessels.

The low numbers of CD8+ cells within malignant nodules, the paucity of granzyme B and CXCR3+ cells, the expression of CXCL12 within stroma and possibly the presence of FoxP3+ putative regulatory cells suggest components of a tumour microenvironment supportive of tumour growth and hostile to immunity. The common pattern of distribution strongly suggested that not only CD8+, but other inflammatory cells were responding to signals associated with the malignant cells in some but not all tumour regions, enabling them to extravasate into the stroma. There they appear to have migrated to the margins of malignant nodules, even penetrating superficially along smaller septa within the tumour masses, yet fail to achieve the cell-cell contact expected for immune-killing.

MCPyV is usually present in MCC [10] in which it expresses tumour antigens now known to contain multiple potential epitope targets for CD8+ and CD4+ effector cells [14]. Patient outcomes appear to associate with intra-tumoural CD8^+^ lymphocytes [16]. This evidence suggests potential for therapeutic vaccination. However, we demonstrate that (i) the inflammatory infiltrate includes significant numbers of macrophages that might exert anti-inflammatory pro-oncogenic effects along with putative regulatory T cells; (ii) T_H_1–polarised interactions or cytolytic effectors are scarce; and (iii) the CD8+ cells line up adjacent to, but excluded from, the malignant nodules. It is clear that MCC T cell therapy will require better understanding of the tumour microenvironment supporting strategies to address interactions hostile to immune effectors.
